# Empowerment of CAR-T Cells by IL-7 and IL-15 Boosts Their Efficacy Against HER2-Positive Tumors with Enhanced Expansion and Persistence

**DOI:** 10.3390/cells15060547

**Published:** 2026-03-19

**Authors:** Zhehong Cheng, Henning Kirchgessner, Beate Jahraus, Emre Balta, Yvonne Samstag

**Affiliations:** 1 Section of Molecular Immunology, Institute of Immunology, Heidelberg University Hospital, 69120 Heidelberg, Germany; zhehong.cheng@stud.uni-heidelberg.de (Z.C.); henning.kirchgessner@immu.uni-heidelberg.de (H.K.); beate.jahraus@immu.uni-heidelberg.de (B.J.); 2 Umbilical Cord Blood, Cell and Tissue Center, STEMBIO, 41400 Kocaeli, Türkiye

**Keywords:** IL-7, IL-15, CAR-T cells, HER2, immunotherapy of cancer

## Abstract

Chimeric antigen receptor (CAR)-T cell therapy has achieved remarkable clinical success in B cell malignancies. However, its efficacy in solid tumors remains limited, in part due to suboptimal expansion, persistence, and restrained effector function. Strategies that promote durable CAR-T cell fitness are therefore required to overcome these barriers. In this study, we generated HER2-CAR-T cells targeting human breast cancer cells and evaluated the impact of different cytokine supplementation strategies on CAR-T cell phenotype and function. We analyzed gene expression patterns and performed repetitive tumor killing assays to assess the ability of CAR-T cells expanded with IL-2 + IL-7 + IL-15 compared with IL-2 alone to maintain proliferation and cytotoxic function across multiple rounds of tumor cell exposure. Compared with IL-2 alone, supplementation with IL-7 and IL-15 significantly enhanced CAR-T cell expansion, preserved stem cell-like features prior to antigen encounter, and promoted superior proliferative capacity. Moreover, CAR-T cells cultured with IL-7+15 or IL-2+7+15 maintained sustained cytotoxicity and exhibited increased antitumor cytokine production during repeated tumor challenges. Notably, IL-7 and IL-15 supplementation induced a CD57^+^ CAR-T cell population that, unlike the immunosenescent CD57^+^ cells reported previously, retained full proliferative and cytotoxic capacity, with CD57 expression being dynamically downregulated upon antigen stimulation. Collectively, these findings demonstrate that incorporation of IL-7 and IL-15 into CAR-T cell manufacturing protocols substantially improves expansion, persistence, and effector function, supporting their use as a strategy to enhance CAR-T cell performance against solid tumors.

## 1. Introduction

The remarkable breakthrough in tumor immunotherapy in recent years has opened new opportunities to cancer patients. Among those immunotherapies, adoptive T cell therapies, such as CAR-T cell therapy, have provided an excellent solution for acute lymphoblastic leukemia [1] and multiple myeloma [2]. However, despite encouraging preclinical results in mouse models and early-phase clinical trials in specific solid tumor contexts, the broader application of CAR-T cell therapies to solid tumors remains challenging and has yet to achieve consistent success [3]. Poor T cell trafficking to tumor sites, an immunosuppressive tumor microenvironment, and most importantly, insufficient activation or proliferation could severely impede the antitumor function of CAR-T cells [4,5,6]. Therefore, the preparation of CAR-T cells with proliferation potential and persistence is crucial for effective CAR-T cell immunotherapy.

Before infusion into patients, T cells transduced with CAR undergo ex vivo cell culture, which is one of the most influential steps determining the functional status of CAR-T cells before application. For CAR-T cell generation, the approach widely applied in FDA-approved clinical products and good manufacturing practices (GMP)-compliant manufacturing protocols relies on common γ-chain cytokines, specifically IL-2 [7,8,9]. IL-2 is a key cytokine regulating T cell proliferation and survival, and was among the earliest cytokines used clinically [10]. However, high doses of IL-2 can also induce T cell exhaustion and terminal differentiation [11]. Conversely, low doses of IL-2 may be insufficient to drive robust T cell expansion, yet can still promote regulatory T cell (T_reg_) expansion [12]. T_reg_ suppress cytotoxic T cells by secreting immunosuppressive cytokines and competitive consumption of IL-2 [12,13], potentially limiting long-term CAR-T cell persistence. Other common γ-chain cytokines, such as IL-7 and IL-15, may be good substitutes or supplements for IL-2. IL-7 signaling is critical for the long-term maintenance of all T cells and promotes survival and homeostasis by preventing T cell apoptosis [14]. IL-15 impacts survival and homeostasis more decisively for CD8^+^ T cells than CD4^+^ cells [15]. It enhances CD8^+^ T cell responses by inhibiting the IL-2-induced activation-induced cell death (AICD) of effector T cells [16]. IL-7 and IL-15 can modulate T cell memory phenotype and differentiation, promoting the development of stem cell memory (T_scm_) and central memory (T_cm_) cell populations [15,17]. Adoptive transfer of T_scm_ or T_cm_ cells has demonstrated improved therapeutic activity due to increased persistence [18]. Nowadays, IL-2, IL-7 or IL-15 have been widely applied in CAR-T cell manufacturing or therapies [19,20,21,22,23], but whether it is preferable to apply the combination of all three cytokines has not been studied yet.

We hypothesized that incorporating all three cytokines into CAR-T cell generation protocols would improve therapeutic outcomes compared to standard IL-2 protocols. Here, we systematically evaluated the effects of IL-2, IL-7 and IL-15, individually or in combination, on HER2-CAR-T cell expansion, differentiation and functional persistence through repetitive tumor challenge. Our comprehensive analysis reveals that the combination of all three cytokines generated CAR-T cells with superior proliferation capacity, increased T_scm_ populations in CD4^+^ cells and sustained antitumor functions, indicated by enhanced antitumor cytokine production and sustained cytotoxicity during repeated tumor challenges. This study provides a mechanistic foundation for CAR-T cell manufacturing, together with in-depth understanding of CAR-T cell phenotypes after repetitive tumor challenge.

## 2. Materials and Methods

### 2.1. Human Peripheral Blood T Cell (PBT) Acquisition

Fresh peripheral blood, which had been heparinized, was collected from healthy volunteers and underwent density gradient centrifugation with FicoLite (LINARIS, Mannheim, Germany, LIN-GTF1511KYA). PBTs were purified using a negative beads selection kit (Miltenyi Biotec, Bergisch Gladbach, Germany, 130-096-535) and kept in RPMI medium (Gibco, Glasgow, UK), supplemented with 10% fetal calf serum (FCS, Gibco) and additional 2 mM glutamine (Gibco) before activation.

### 2.2. Cell Lines and Cell Culture

HER2-positive SKBR-3 and MDA-MB-453 cells were purchased from the German Collection of Microorganisms and Cells (DMSZ, Braunschweig, Germany) and cultured in Dulbecco’s modified Eagle medium (DMEM, Sigma, Setagaya City, Japan) with 10% FCS. All cells were kept in incubators at 37 °C with humidity and 5% CO_2_.

### 2.3. Lentivirus Production and CAR-T Cell Production

The construct of HER2-CAR and Control (Ctrl)-CAR, the virus production as well as CAR-T cell production were described previously [24]. Briefly, PBTs were activated by anti-CD3/CD28 beads (Gibco, 11161D) for 24 h in X-vivo 15 medium (LONZA, Walkersville, MD, USA), according to the product manual. Afterwards, cells were cultured with lentiviral vectors, 20 U/mL IL-2 (2 ng/mL, Thermo Fisher Scientific, Waltham, MA, USA, 200-02), 10 U/mL IL-7 (10 ng/mL, Thermo Fisher Scientific, 200-07), 10 U/mL IL-15 (10 ng/mL, Thermo Fisher Scientific, 200-15) and polybrene. Spinoculation was used to increase transduction efficiency. Seventy-two h after transduction, pseudoviral particles were discarded by centrifugation and repetitive washing. CAR-T cells were then maintained in X-vivo 15 medium with the above interleukins until 8 days after activation. Then, anti-CD3/CD28 beads were removed, and cells were transferred into complete RPMI medium with the above interleukins. The CAR receptor expression level was measured by flow cytometry (Symphony A3, BD Biosciences, Franklin Lakes, NJ, USA) using PE-conjugated goat anti-human immunoglobulin G (IgG) antibody (Novus Biologicals, Wiesbaden, Germany, NBP1-75002PE, RRID: AB_3218911).

### 2.4. Measurement of CAR-T Cell Expansion

For quantification of viable CAR-T cells, the total volume of cell suspension was first recorded. An aliquot of 100 μL of the cell suspension was then stained with 7-AAD viability dye (1:50, BD Biosciences, 420404) and anti-CD3 FITC antibody (1:100, BD Biosciences, 345763, RRID: AB_2811220) for 20 min. Subsequently, 10 μL of Precision Count Beads (~1 × 10^6^ beads/mL, BioLegend, San Diego, CA, USA 424902) was added. Samples were then immediately analyzed by flow cytometry. The ratio of 7-AAD-viable CD3^+^ CAR-T cells to Precision Count Beads was used to calculate the absolute cell density of the original cell suspension.

### 2.5. nCounter mRNA Analysis

CAR-T cells were prepared and activated as described above. Eight days after activation, the cell culture medium was replaced by serum-free culture medium and cells were cultured for another 6 h. Next, total RNA was isolated using RNA isolation kits (Zymo Research, Freiburg, Germany, R2051). RNA yield and integrity were assessed using a NanoDrop spectrophotometer (Thermo Fisher Scientific) and bioanalyzer (Agilent Technologies, Santa Clara, CA, USA), respectively. Gene expression profiling was conducted using a NanoString nCounter Immune Exhaustion Pathway panel. Data processing and analysis were performed as previously described [25]. mRNA code counts in each sample were extracted using nSolver Analysis Software Version 4.0 (NanoString Technologies, Bothell, WA, USA).

### 2.6. Flow Cytometry-Based Cytotoxicity Assay

The flow cytometry-based cytotoxicity assay was established to evaluate the killing capacity of HER2-CAR-T cells. SKBR-3 cells were seeded to 48-well plates (5 × 10^4^ per well) and adhesion was allowed overnight. The next day, CAR-T cells with different interleukin settings were co-incubated with tumor cells at different effector–target ratios (E:T ratios) in 500 μL of complete RPMI medium. After 24 h of incubation, the supernatant with suspending CAR-T cells was discarded, and tumor cells were washed by PBS once. The tumor cells then underwent trypsinization at room temperature to detach. Cells were washed by PBS twice and stained with eF780 viability dye (Thermo Fisher Scientific, 65-0865-18) and anti-HER2-PE antibody (1:500, BioLegend, 324406, RRID: AB_756122) for 20 min. After washing in FACS wash buffer (PBS + 1% BSA) three times, 10 μL of Precision Counting Beads was added to the cell suspension, and the mixture was measured immediately by flow cytometry. Relative living tumor cell numbers were calculated by dividing the cells/beads ratio of each group by the ratio of the tumor cell-only control.

### 2.7. Repetitive Tumor Killing Assay

SKBR-3 cells were seeded in 48-well plates at a density of 5 × 10^4^ per well and incubated overnight. On the following day (9 days after initial anti-CD3/28 activation), 2 × 10^5^ CAR-T cells were cocultured with tumor cells in 500 μL complete RPMI medium with different interleukins for 4 days. CAR-T cells were collected and re-cocultured with the tumor cells at the same density for another 4 days.

### 2.8. Magnetic Separation of CD57^+^ CAR-T Cells

After activation and transduction, HER2-CAR-T cells were expanded with IL-2+7+15 and kept for 15 days, when an obvious CD57^+^ population could be observed. The magnetic separation of CD57^hi^ and CD57^lo^ cells was conducted using anti-CD57 magnetic beads (Miltenyi Biotec, 130-092-073) and a QuadroMACS™ separator (Miltenyi Biotec, 130-090-976) according to the manufacturer’s instruction. Sorted cells were kept in RPMI medium with interleukins overnight and then used in repetitive tumor killing assays by co-incubation with SKBR-3 tumor cells.

### 2.9. Cell Proliferation Tracking

The cells were washed twice with PBS and adjusted to a concentration of 1 million/100 µL in PBS. Tag-it Violet (BioLegend, 425101) staining solution was added. The working concentration of cell tracking dye was 0.25 µM and the cells were incubated for 10 min at 37 °C. Cells were then washed by complete RPMI medium and centrifuged. Cell pellets were resuspended in 5 mL complete RPMI and were left for 30 min at 37 °C. The cells were again centrifuged and collected. They were finally cultured in complete RPMI with interleukins at a concentration of 400,000 cells/mL. 

### 2.10. Immune Synapse Formation

HER2-CAR-T cells were expanded with IL-2+7+15 for 15 days. MDA-MB-453 tumor cells were detached using PBS + 5 mM EDTA for 3 min. Next, CAR-T cells and MDA-MB-453 cells were washed with complete RPMI medium. Then, 4 × 10^5^ CAR-T cells and 2 × 10^5^ MDA-MB-453 cells were mixed in round-bottom polystyrene tubes in 200 μL complete RPMI for 15 or 45 min, followed by gradual addition of 2 mL 1.5% paraformaldehyde (PFA) onto the samples while they were gently vortexed to prevent non-specific interactions. Samples were fixed for 10 min at room temperature, followed by permeabilization in PBS with 1% bovine serum albumin (BSA) and 0.1% saponin for 10 min. Finally, samples were stained with rabbit anti-phosphorylated L-plastin (pLPL) mAb (1:1500, produced by us) followed by donkey anti-rabbit IgG-AF488 (1:2500, Jackson ImmunoResearch, West Grove, PA, USA, 711-546-152, RRID: AB_2340619), CD57-PE (1:100, Miltenyi Biotec, 130-111-810, RRID: AB_2658748), CD3-PE-Cy7 (1:25, BD, 557851, RRID: AB_396896), HER2-BV421 (1:50, BioLegend 324420, RRID: AB_2563990), and Phalloidin-AF647 (1:250, Invitrogen, Waltham, MA, USA, A22287). The synapse formation was measured by ImageStream^X^ MK II imaging flow cytometry (Cytek, Fremont, CA, USA) and analyzed by IDEAS v. 6.4.6.0.

### 2.11. Cytokine Release Assay

Six hours after starting each flow cytometry-based cytotoxicity assay, supernatants from cocultured cells or tumor cells alone (control) were collected by centrifugation and kept at −20 °C. The release of cytokines was measured using the LegendPlex Human CD8/NK Panel kit (BioLegend, 741186) according to the manufacturer’s manual. The analysis was performed by BioLegend Qognit (v. 2025-05-01).

### 2.12. Phenotypical Characterization of CAR-T Cells

CAR-T cells cultured alone or cocultured with tumor cells were collected by centrifugation. Cells were washed once with PBS and stained to evaluate their phenotype. The following antibodies were used for staining: (i) memory phenotyping, CD4-AF700 (BioLegend, 357418, RRID: AB_2616933), CD8-BV605 (BioLegend, 344741, RRID: AB_2566512), CD95-PE (BioLegend, 305608, RRID: AB_314546), CD45RA-APC (BioLegend, 304112, RRID:AB_314416), CCR7-PE-Cy7 (BioLegend, 353226, RRID: AB_11126145), CD62L-BV510 (BioLegend, 304844, RRID: AB_2617003), CD57-Pacific Blue (BioLegend, 359608, RRID: AB_2562459), Granzyme B-FITC (BD Biosciences, 560211, RRID: AB_1645488); (ii) exhaustion phenotyping, CD3-PE (BD Biosciences, 345765, RRID: AB_2868796), CD4-AF700, CD8-FITC (BD Biosciences, 555366, RRID: AB_395769), PD1-APC (BioLegend, 367406, RRID: AB_2566067), TIM-3-BV421 (BioLegend, 345008, RRID: AB_11218598), LAG3-PE-Cy7 (BioLegend, 369310, RRID: AB_2629753) according to the manufacturer’s instructions.

### 2.13. Data Analysis

The flow cytometry data were analyzed by BD Flowjo v. 10. The statistical analyses were performed by Graphpad Prism v. 10.

## 3. Results

### 3.1. Compared to Cultivation in IL-2 Alone, Cultivation with IL-2+7+15 Led to Improved CAR-T Cell Expansion

**Figure 1:**
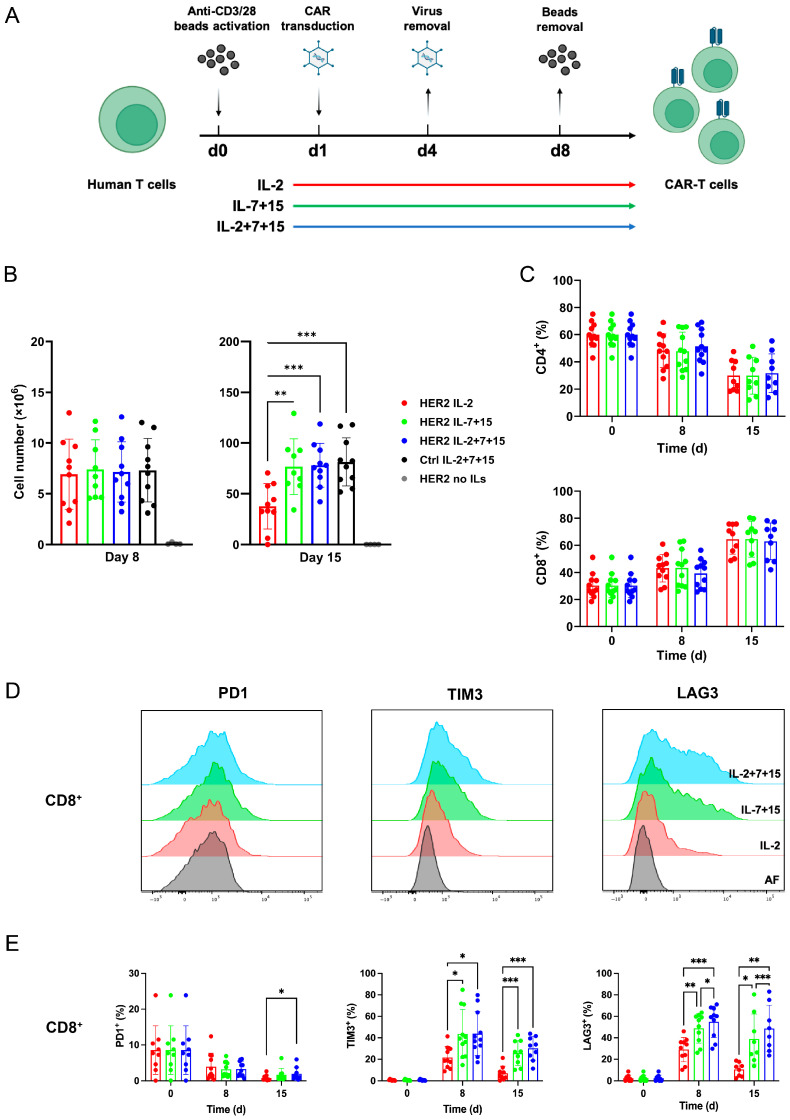
Compared to cultivation in IL-2 alone, cultivation with IL-2+7+15 led to improved CAR-T cell expansion and enhanced expression of TIM3 and LAG3. (**A**) Schematic description of CAR-T cell generation. Purified fresh T cells from healthy donors were activated. On the following day, the cells were transduced with lentiviral vectors for 72 h. Different interleukin settings were applied to the cells in the period subsequent to viral transduction. (**B**–**E**) After removal of the anti-CD3/28 beads, CAR-T cells were further kept in medium with different interleukins until 15 days after initial activation. (**B**) Expansion of CAR-T cells with different interleukins. Live T cells were counted on day 8 and day 15 by flow cytometry. Ctrl: Ctrl-CAR-T cells. No ILs: CAR-T cells cultured without interleukin addition. (**C**) CD4^+^ and CD8^+^ proportion of CAR-T cells. (**D**) Histogram of PD1, TIM3 and LAG3 expression of CD8+ HER2-CAR-T cells on day 8. AF: autofluorescence. (**E**) Exhaustion phenotype of CD8^+^ CAR-T cells. Statistical analyses were done by matched ANOVA by donors. *: *p* < 0.05; **: *p* < 0.01; ***: *p* < 0.001.

To investigate how IL-2, IL-7 and IL-15 influence the proliferation and phenotype of CAR-T cells, we applied different interleukins during CAR-T cell generation (Figure 1A, see Section 2.3 for detail). Peripheral blood T cells from healthy donors were isolated, activated by anti-CD3/CD28 stimulation, and transduced with lentiviral particles encoding for the HER2-CAR construct or the non-functional Control (Ctrl-CAR) construct (which cannot target HER2). The following were added to the CAR-T cells from the start of the transduction: IL-2 alone; IL-7 and IL-15 (hereafter referred to as IL-7+15); and combinations of IL-2, IL-7 and IL-15 (IL-2+7+15). CAR-T cell expansion was analyzed by cell counting on day 8 and day 15 after the first encounter of anti-CD3/28 bead activation. On day 8 (Figure 1B, left), the number of CAR-T cells obtained after treatment with the different interleukins reached a similar level of about 8 million cells derived from 80 thousand original cells on average, except the group with no addition of interleukins (referred to as no ILs). In contrast, on day 15 (Figure 1B, right), CAR-T cells cultivated in the presence of IL-7+15 or IL-2+7+15 expanded significantly more than those cultivated with IL-2 only to about 80 million for IL-7+15 or IL-2+7+15, as compared to about 40 million for IL-2 only. Thus, between day 8 and day 15, the number of cells with IL-7+15 and IL-2+7+15 increased by about ten times, while cells with IL-2 increased only by five times. This finding demonstrates that IL-7 together with IL-15 markedly increased T cell proliferation beginning on day 8. The percentage of CD4^+^ CAR-T cells decreased and the percentage of CD8^+^ CAR-T cells increased over the 15-day culture period. This shift in the CD4^+^/CD8^+^ ratio was consistent across all cytokine conditions, indicating that interleukin treatment did not differentially influence the CD4^+^/CD8^+^ composition of the CAR-T cell product (Figure 1C).

### 3.2. Compared to IL-2 Alone, Cultivation with IL-2+7+15 Enhanced the Expression of TIM3 and LAG3, but Not PD1, in HER2-CAR-T Cells

We next measured the exhaustion phenotype of the HER2-CAR-T cells. For the T cell exhaustion marker PD1, in CD8^+^ T cells on day 15 (Figure 1D,E, left), not in CD4^+^ T cells (Figure S1), a slightly higher percentage of IL-2+7+15-treated CAR-T cells expressed PD1 than in IL-2-treated CAR-T cells. Although this increase in CD8^+^ cells reached statistical significance, the absolute difference was small (day 15, IL-2 vs. IL-2+7+15: approximately 0.8% vs. 1.9%). In contrast, IL-7+15- and IL-2+7+15-treated CAR-T cells had a significantly higher expression of TIM3 (Figure 1D,E, middle) and LAG3 (Figure 1D,E, right) than IL-2-treated cells in both CD8^+^ (Figure 1D,E) and CD4^+^ (Figure S1) CAR-T cells on day 8 and day 15 after costimulation via CD3/CD28. Note that the percentage of PD1-, TIM3- and LAG3-expressing cells slightly dropped on day 15, compared with day 8.

### 3.3. Compared to IL-2 Alone, Cultivation with IL-2+7+15 Resulted in an Increase in the Stem Cell Memory CD4+ CAR-T Cell Population and Enhanced Tscm-Associated Gene Expression

**Figure 2:**
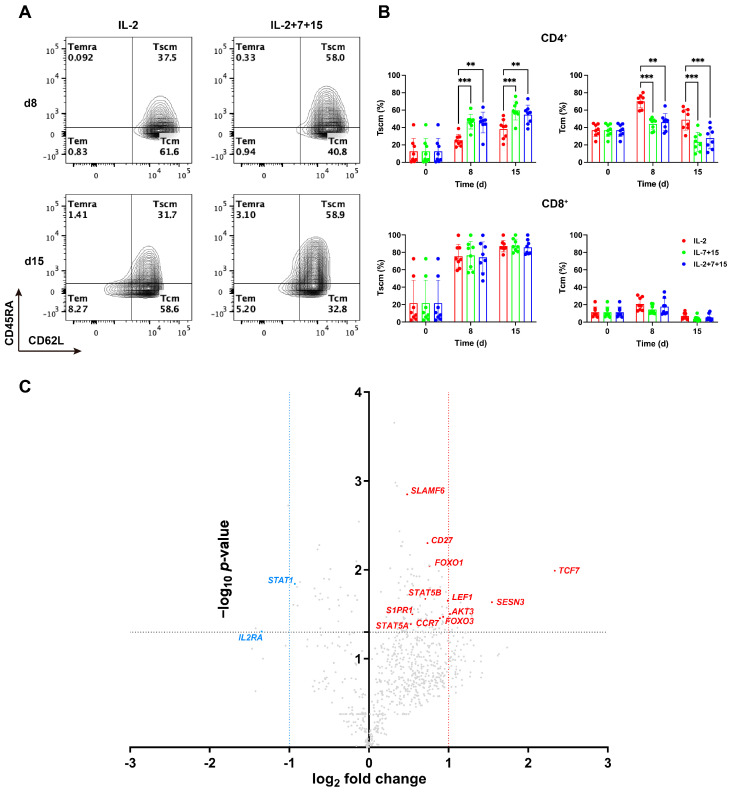
Compared to IL-2 alone, cultivation with IL-2+7+15 resulted in an increase in the stem cell memory CD4+ CAR-T cell population and enhanced T_scm_-associated gene expression. (**A**) Representative contour plot of the memory phenotype of CD4^+^ CAR-T cells on day 8 and day 15 after initial anti-CD3/CD28 activation. T_scm_: CD62L^+^CD45RA^+^CD95^+^; T_cm_: CD62L^+^CD45RA^−^; T_em_: CD62L^−^CD45RA^−^; T_emra_: CD62L^−^CD45RA^+^. (**B**) Statistical evaluation of memory phenotype of HER2-CAR-T cells based on CD62L and CD45RA expression on day 8 and day 15 after initial anti-CD3/CD28 activation. (**C**) Volcano plot showing log-transformed gene expression comparison at mRNA level of HER2-CAR-T cells cultured with IL-2 alone or with IL-2+7+15 on day 8 (*n* = 3). Key genes upregulated or downregulated in IL-2+7+15-treated cells were marked as red or blue, respectively. The statistical analysis in (**B**) was done by matched ANOVA by donors. **: *p* < 0.01; ***: *p* < 0.001.

The memory phenotype of the differently treated CAR-T cells was determined by flow cytometry according to their expression of CD62L, CD45RA, and CD95 on the cell surface (gating strategy in Figure S2). Under IL-7+15 and IL-2+7+15 conditions, the T_scm_ and T_cm_ populations (Figure 2A,B) dominated the memory phenotype in both CD4^+^ and CD8^+^ T cells from 8 days of CAR-T cell expansion onwards. The percentage of effector memory CD62L^−^CD45RA^−^ T cells (T_em_) and terminally differentiated CD62L^−^CD45RA^+^ T cells (T_emra_) in both CD4^+^ and CD8^+^ cells was found to be low (combined, less than 10%), and significant differences between treatments were not observed (Figure S3). Interestingly, a significant increase in the CD62L^+^CD45RA^+^CD95^+^ T_scm_ populations in IL-7+15- and IL-2+7+15-treated cells was observed, when compared to IL-2-treated cells. This increase was on average more than 50%. However, it was only observed in the CD4^+^ (Figure 2A,B, upper panel), but not in the CD8^+^ CAR-T cell population (Figure 2B, lower panel). Accordingly, the CD62L^+^CD45RA^−^ T_cm_ population was significantly higher in IL-2-treated cells, only in the CD4+ CAR-T cell population (Figure 2A,B). Taken together, IL-7+15- and IL-2+7+15-treated CAR-T cells proliferated stronger and contained a larger population of CD4^+^ T_scm_.

We further addressed the gene expression profile of CAR-T cells cultured with IL-2 or IL-2+7+15. To this end, we employed the nCounter^®^ Immune Exhaustion Panel, allowing real-time quantification of 785 genes at the mRNA level (Figure 2C, Table S1). Importantly, most of the genes that were upregulated in IL-2+7+15-treated CAR-T cells on day 8 were related to T cell persistence and the T_scm_ state (marked in red). These included *CD27*, *FOXO1* and *TCF7*. Specifically, *TCF7* was the gene that was most highly upregulated in CAR-T cells cultured with IL-2+7+15 compared to CAR-T cells cultured with IL-2 only. These data provided strong evidence for the improved stem cell properties and functionality of CAR-T cells after cultivation with IL-2+7+15.

### 3.4. HER2-CAR-T Cells Expanded More Effectively upon Repetitive Tumor Challenges in the Presence of IL-2+7+15 than in the Presence of IL-2 Alone, Particularly with Regard to CD4+ Cells

**Figure 3:**
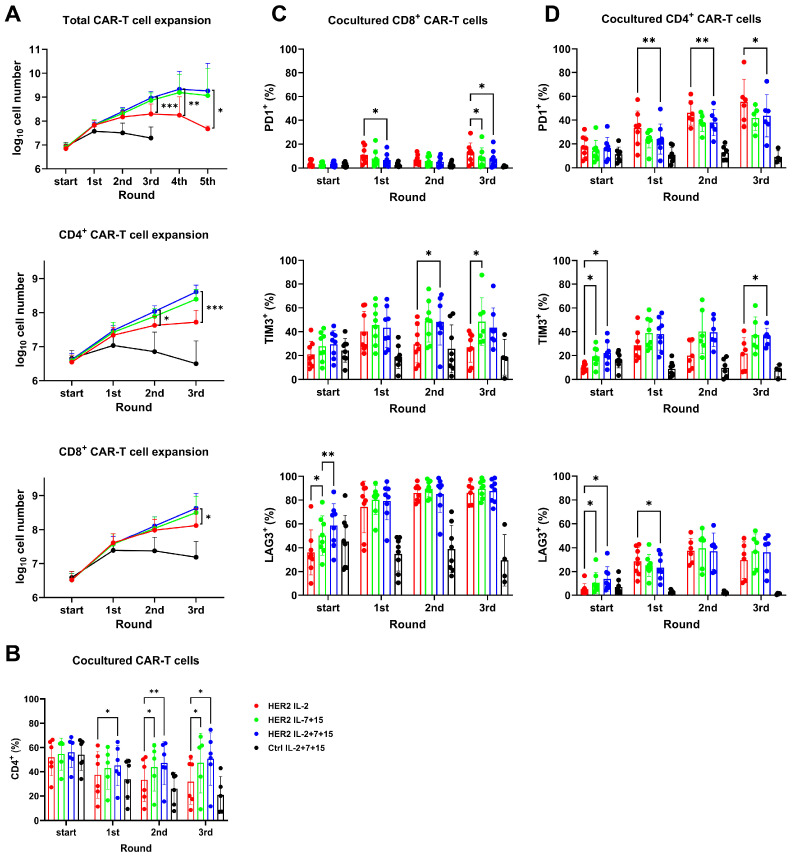
HER2-CAR-T cells expanded more effectively upon repetitive tumor challenges in the presence of IL-2+7+15 than in the presence of IL-2 alone, particularly with regard to CD4+ cells. Nine days after initial anti-CD3/CD28 activation (start), CAR-T cells were cocultured with SKBR-3 tumor cells at the ratio of 4:1, with different interleukins. Every 4 days, cells were counted, stained for phenotyping, and proceeded to another round of coculture. (**A**) Expansion of CAR-T cells during repetitive killing. The total CAR-T cell expansion and CD4^+^/CD8^+^ CAR-T cell expansion were measured by cell counting. Group comparison was conducted between IL-2-treated cells and IL-2+7+15-treated cells. (**B**) The percentage of CD4^+^ T cells within total CAR-T cells after each round of tumor exposure. (**C**) Exhaustion phenotype of cocultured CD8^+^ CAR-T cells. (**D**) Exhaustion phenotype of cocultured CD4^+^ CAR-T cells. Group comparisons in (**B**–**D**) were conducted between HER2-CAR-T cells with different treatments. Statistical analyses were done by matched ANOVA by donors. *: *p* < 0.05; **: *p* < 0.01; ***: *p* < 0.001.

**Figure 4:**
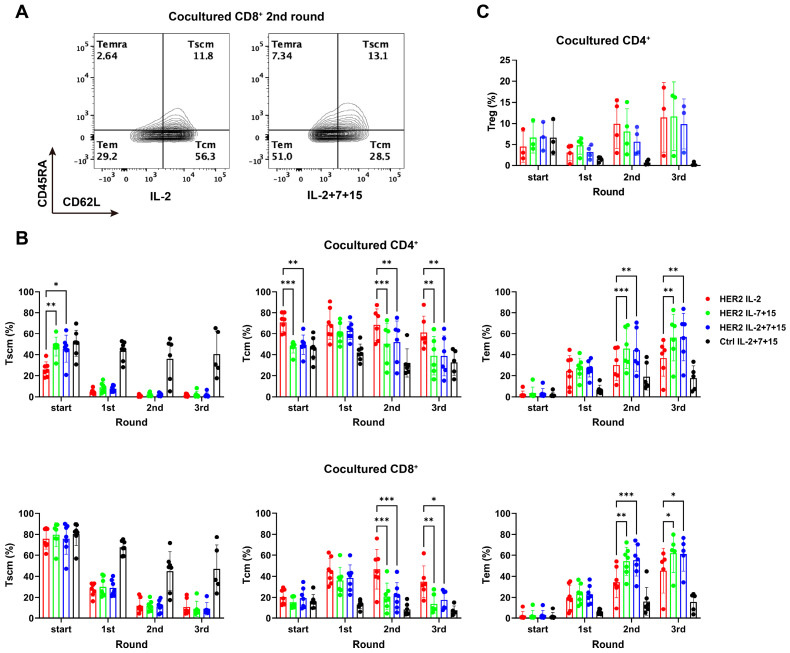
IL-2+7+15 treatment increased the differentiation of HER2-CAR-T cells to T_em_ after tumor challenge, compared to IL-2 alone. During each round of the repetitive tumor killing assay, the memory phenotype of CAR-T cells cocultured with tumor cells was analyzed. (**A**) Representative contour plot of memory phenotype of CD8^+^ T cells after the second round of tumor cell coculture. T_scm_: CD62L^+^CD45RA^+^CD95^+^, T_cm_: CD62L^+^CD45RA^−^. T_em_: CD62L^−^CD45RA^−^, T_emra_: CD62L^−^CD45RA^+^. (**B**) Statistical evaluation of memory phenotypes in CD4^+^ and CD8^+^ CAR-T cells. (**C**) T_reg_ proportion in CD4^+^ CAR-T cells. Group comparisons were conducted between HER2-CAR-T cells with different treatments. Statistical analyses were done by matched ANOVA. *: *p* < 0.05; **: *p* < 0.01; ***: *p* < 0.001.

Next, we conducted repetitive tumor killing assays to explore whether CAR-T cells with IL-2+7+15 could withstand tumor exposure and keep proliferating. In this assay, HER2-positive SKBR-3 cells were used to provide antigen-specific stimulation. CAR-T cells expressing either the HER2-CAR receptor or non-functional Ctrl-CAR receptor were cocultured with tumor cells from day 9 on (marked as start in Figure 3 and Figure 4) for multiple rounds.

As shown by total cell counting (Figure 3A, upper panel), HER2-CAR-T cells maintained proliferation upon repetitive antigen-specific stimulation, with this maintenance occurring across all interleukin conditions. In contrast, Ctrl-CAR-T cells, which lacked antigen-specific stimulation, ceased to proliferate and slowly died. Therefore, they could not be used for coculture beyond the third round of tumor cell exposure. From the third round of coculture on, HER2-CAR-T cells supported by IL-2+7+15 expanded significantly more than IL-2-only treated HER2-CAR-T cells. The IL-7+15-treated HER2-CAR-T cells and IL-2+7+15-treated HER2-CAR-T cells shared similar proliferation profiles. Cell numbers of IL-7+15- and IL-2+7+15-treated HER2-CAR-T cells reached the plateau at the fourth exposure. IL-2-treated cells largely ceased to expand by the third round, and there were significantly fewer of them than of the IL-7+15- and IL-2+7+15-treated cells. By the fourth round, the number of IL-2-treated cells had even dropped by a factor of 10 compared to IL-7+15- and IL-2+7+15-treated cells.

Separate analysis of the percentage of CD4^+^ versus CD8^+^ HER2-CAR-T cells showed a significant decline in the ratio of CD4^+^ HER2-CAR-T cells in the absence of IL-7 and IL-15 (Figure 3B). Accordingly, the absolute CD4^+^ T cell counts (Figure 3A, middle) diverged significantly after the second round of tumor challenge—preceding differences in total CD8^+^ CAR-T cell numbers (Figure 3A, lower panel). Together, these findings indicated that IL-7 and IL-15 supported CAR-T cell proliferation preferentially during repetitive antigen stimulation, particularly within the CD4^+^ subset.

Next, the exhaustion profile of CAR-T cells during the repetitive tumor killing was analyzed by flow cytometry. Although IL-2-treated CD8+ HER2-CAR-T cells showed slightly higher PD1 expression than IL-7+15- and IL-2+7+15-treated cells, the overall PD1^+^ percentage of CD8^+^ HER2 CAR-T cells stayed at a level below 20% on average (Figure 3C, upper panel). In contrast, CD4^+^ HER2-CAR-T cells expressed PD1 at higher levels than CD8^+^ cells across all treatment groups from the first challenge on and the level increased continuously in all treatments. Importantly, IL-2-treated HER2-CAR-T cells exhibited significantly higher PD1 expression than IL-2+7+15-treated cells in the CD4^+^ population from the first challenge onwards (Figure 3D, upper panel). Both CD8^+^ (Figure 3C) and CD4^+^ (Figure 3D) HER2-CAR-T cells showed higher TIM3 expression in IL-7+15- and IL-2+7+15-treated HER2-CAR-T cells than IL-2-treated cells after two or three rounds of tumor killing, but their LAG3 levels were similar. Without antigen-specific stimulation, the exhaustion phenotype of Ctrl-CAR-T cells (black bars) remained mostly unchanged.

### 3.5. IL-2+7+15 Treatment Increased the Differentiation of HER2-CAR-T Cells to Tem After Tumor Challenge Compared to IL-2 Alone

We next examined changes in the memory phenotype of CAR-T cells across successive rounds of antigen exposure. As shown in Figure 4A,B, antigen recognition on HER2-positive SKBR3 tumor cells dramatically reduced the T_scm_ population of HER2-CAR-T cells for all treatments after the first round of tumor killing, while promoting T_em_ populations (Figure 4A,B). Without antigen-specific stimulation, Ctrl-CAR-T cells largely maintained their T_scm_ population (Figure 4B, left). Whereas the memory phenotype of HER2-CAR-T cells treated with the different interleukins was similar after the first tumor exposure, from the second tumor exposure on, the T_cm_ proportion of both CD4^+^ and CD8^+^ populations decreased (Figure 4B, middle), while the T_em_ proportion continued to increase (Figure 4B, right). Thereby, HER2-CAR-T cells with IL-7+15 or IL-2+7+15 showed significantly higher increases in T_em_ and greater decreases in T_cm_, as compared to HER2-CAR-T cells with IL-2 only. Following the third tumor exposure, the proportion of T_em_ in IL-7+15 or IL-2+7+15 HER2-CAR-T cells reached approximately 60%, which was 20% higher than that of IL-2 HER2-CAR-T cells.

To determine if the mere interleukin treatment influenced the memory phenotype, independently of antigen stimulation, we measured in parallel the phenotype for CAR-T cells that were cultured in the absence of tumor cells (Figure 4, w/o tumors). Here, in contrast to tumor-exposed CAR-T cells, the memory phenotype of both CD4^+^ and CD8^+^ cells remained the same as was seen during CAR-T cell generation (compare Figure 2B). Specifically, the T_scm_ populations of IL-7+15- and IL-2+7+15-treated CD4^+^ cells were larger than in IL-2-only treated cells. Under all interleukin conditions, the T_scm_ population slightly increased with time, while the T_cm_ population decreased. In contrast to tumor-exposed CAR-T cells, only a few T_em_ cells were generated.

Since the presence of T_reg_ may harm the antitumor response of CAR-T cells, it was important to determine whether T_reg_ expansion was differentially influenced by the different interleukin treatments. As seen in Figure 4C (start), after 9 days of CAR-T cell expansion, the percentage of CD4^+^CD25^+^Foxp3^+^ T_reg_ within the total CD4^+^ HER2-CAR-T cells treated with different interleukins was equal (approximately 5% on average). Following the second and third tumor exposure, the T_reg_ population increased to approximately 10% of CD4^+^ HER2-CAR-T cells, while it rather decreased in Ctrl-CAR-T cells, namely in the absence of tumor cell stimulation. Importantly, IL-2+7+15 treatment did not enrich the T_reg_ population as compared to IL-2 treatment alone, both before or after tumor exposure.

Together, these findings demonstrated that IL-7 and IL-15 improved CAR-T cell expansion and durability during repetitive tumor killing, accompanied by increased T_em_ populations and decreased T_cm_ populations.

### 3.6. Treatment with IL-15 Induced a Population of CD57+ CAR-T Cells Whose CD57 Expression Was Downregulated upon Tumor Challenge

**Figure 5:**
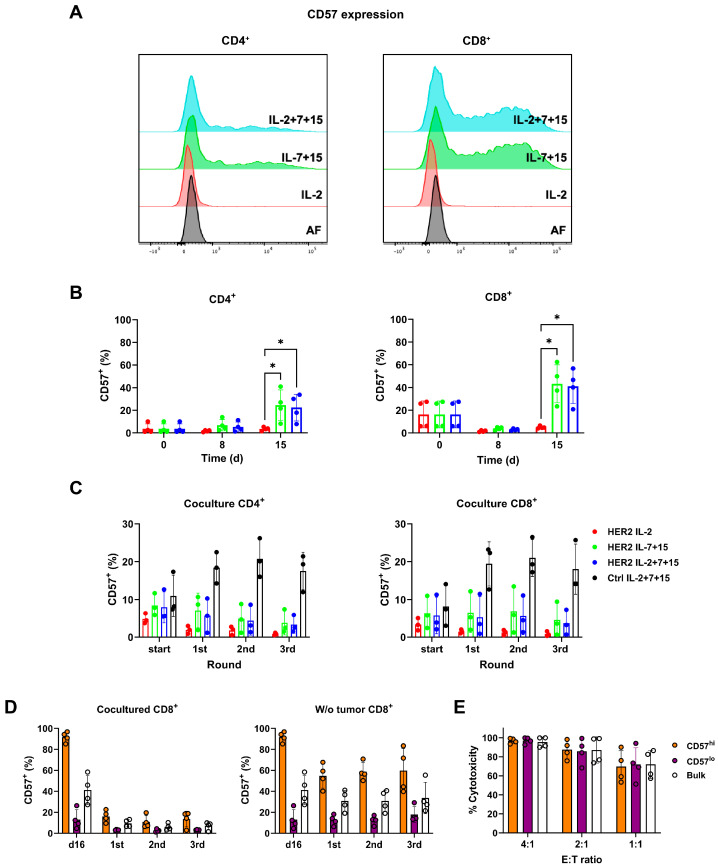
Treatment with IL-15 induced a population of CD57^+^ CAR-T cells, whose CD57 expression was downregulated upon tumor challenge. (**A**,**B**) CD57 expression level of CD4^+^ and CD8^+^ CAR-T cells with different interleukin treatments during CAR-T cell expansion measured by flow cytometry. (**A**) Representative histogram of CD57 expression on day 15 after initial anti-CD3/CD28 activation of CD4^+^ and CD8^+^ CAR-T cells. (**B**) Statistical evaluation of CD57 expression on day 8 and day 15 by matched ANOVA by donors. *: *p* < 0.05. (**C**) Nine days after the initial anti-CD3/28 activation (start), CAR-T cells were used for the repetitive tumor killing assay. Before each round of tumor killing, the CD57 level of CD4+ and CD8^+^ CAR-T cells was measured by flow cytometry. Group comparison of CD57 expressions was conducted between HER2-CAR-T cells with different treatments. (**D**) IL-2+7+15 HER2-CAR-T cells were sorted by anti-CD57 magnetic beads on day 15 after activation. On day 16, the positively sorted CD57^hi^ CAR-T cells, the remaining CD57^lo^ cells, and bulk CAR-T cells were proceeded to a repetitive tumor killing assay for three rounds. Before each round of tumor killing, the CD57 level of CD8^+^ cells was measured. (**E**) On day 16, CD57^hi^, CD57^lo^ and bulk CAR-T cells were used to kill tumor cells at different effector–target (E:T) ratios. CAR-T cells were co-incubated with tumor cells for 24 h. Remaining live tumor cells and HER2 expression were identified by flow cytometry.

CD57 has been reported as a surface marker for immunosenescent CD8^+^ T cells [17,26], which have low self-renewal potential and were thought to be associated with terminal differentiation. Therefore, we measured the level of CD57 during CAR-T cell expansion. A CD57^+^ subpopulation emerged in both CD4^+^ and CD8^+^ CAR-T cells after cultivation for 15 days with IL-7+15 or IL-2+7+15 treatment (Figure 5A,B). As seen in Figure 5B, on day 15, the CD57^+^ population increased to near 40% in CD8^+^ HER2-CAR-T cells and 20% in CD4^+^ HER2-CAR-T cells. In contrast, IL-2-treated CAR-T cells showed no increase in CD57. To further investigate which interleukins contributed to CD57 upregulation, more combinations of interleukins, including the combination of IL-2 and IL-7 and the combination of IL-2 and IL-15, were evaluated (Figure S5). IL-2 alone or a combination of IL-2 and IL-7 were not sufficient. Increased CD57 expression was observed only in cytokine conditions that included IL-15, suggesting that IL-15 is required for this phenotype under the tested conditions. However, whether IL-15 alone is sufficient to induce CD57 expression remains to be determined.

Surprisingly, the CD57^+^ population decreased in interleukin-treated HER2-CAR-T cells cocultured with tumor cells (Figure 5C) but remained present in Ctrl-CAR-T cells (Figure 5C, black bars), which were not activated by the tumor cell coculture. Thus, the repetitive tumor killing assay indicated that antigen-specific stimulation led to a reduction in the CD57^+^ cell population. To further reveal the relationship between antigen stimulation and CD57 expression, 15 days after activation, HER2-CAR-T cells generated with IL-2+7+15 were separated into CD57^hi^ and CD57^lo^ cells by magnetic sorting using anti-CD57 beads. The different populations, CD57^hi^, CD57^lo^, and bulk CAR-T cells, were used to perform another repetitive tumor killing assay. To exclude potential artifacts caused by the sorting procedure, sorted cells were rested overnight and CD57 expression was re-measured prior to functional assays. The CD57^hi^ population retained >90% CD57 positivity after the resting period (Figure 5D), confirming the stability of the sorted populations. CD8^+^ T cells were analyzed since CD57 was more enriched in CD8^+^ T cells based on the previous results. Intriguingly, after even one antigen exposure, CD57 expression on the CD57^hi^ cell population dropped from almost 100% positive to below 20% (Figure 5D, left). This dramatic downregulation of CD57 also happened in bulk CAR-T cells. In cells not exposed to tumor cells (Figure 5D, right, w/o tumor), there was a much smaller decrease in the CD57^+^ population for both CD57^hi^ and bulk CD8^+^ HER2-CAR-T cells. In order to analyze whether CD57 expression would be restored following antigen withdrawal, some of the HER2-CAR-T cells were separated after the initial tumor exposure and cultured without tumor cells to rest for an additional four days. As shown in Figure S6, their CD57 expression did not recover.

To determine the mechanism underlying the reduction in the CD57^+^ population after antigen stimulation, we evaluated whether this change reflected the proliferative dilution, selective cell loss, or modulation of CD57 surface expression. To assess this, both bulk CAR-T cells and CD57^hi/lo^ cells were labeled with a proliferation dye to track their division during the first round of the repetitive tumor killing assay, and total cell numbers were quantified accordingly (Figure S7). Proliferation analyses revealed comparable expansion of CD57^hi^, CD57^lo^, and bulk CAR-T cells, arguing against preferential proliferation of CD57^−^ cells as a cause of the observed shift. In addition, we did not detect a reduction in absolute cell numbers within the CD57^hi^ population, making activation-induced cell death unlikely. Together, these findings indicate that the decrease in the CD57^+^ fraction is best explained by the antigen-driven downregulation of CD57 surface expression rather than selective loss of CD57-expressing cells.

Given that CD57 has been reported as a surface marker for immunosenescent CD8^+^ T cells [17,26], we also performed a tumor killing assay within 24 h to measure the tumor killing capacity of CD57^hi^ versus CD57^lo^, or bulk HER2-CAR-T cells (Figure 5E). However, no significant difference was found in their cytotoxicity against HER2-positive SKBR3 cells.

It was also reported that CD57 expression positively correlated with glucose transporter 1 (GLUT1) and a higher CD57 expression could indicate a different metabolic phenotype [27]. Therefore, we measured the GLUT1 expression but did not find a significant difference in GLUT1 expression between CD57^hi^ versus CD57^lo^, or bulk HER2-CAR-T cells (Figure S8). This result suggests that CD57^+^ CAR-T cells in our model do not display the GLUT1-associated metabolic phenotype described in that report. Broader metabolic profiling (e.g., mitochondrial mass, membrane potential, glycolytic flux measurements) could provide additional insights and will be of interest for future investigations.

To summarize, IL-2+7+15 treatment induced a CD57^+^ subpopulation during long-term culture. This CD57^hi^ subset displayed proliferation and cytotoxicity comparable to CD57^lo^ cells, yet its CD57 expression was strongly downregulated upon antigen stimulation.

### 3.7. CD57 Did Not Localize to the Immunological Synapses Between CAR-T Cells and Tumor Cells

The disappearance of CD57 on HER2-CAR-T cells following tumor cell exposure intrigued us to study whether CD57 played a role in CAR-T cell immune synapse formation, potentially followed by the downmodulation of CD57 from the cell surface. To study this, we introduced an interaction model that allowed the analysis of immunological synapses formed between CAR-T cells and HER2^+^ MDA-MB-453 tumor cells. After the synapses were formed, cells were fixed and stained and colocalization with other cell surface molecules was analyzed by imaging flow cytometry.

**Figure 6:**
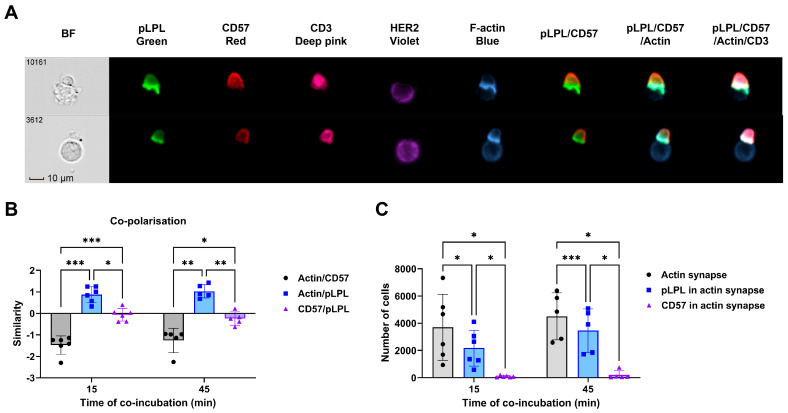
CD57 did not localize to the immunological synapses between CAR-T cells and tumor cells. Fifteen days after initial anti-CD3/28 activation, HER2-CAR-T cells expanded with IL-2+7+15 were cocultured with MDA-MB-453 cells for 15 or 45 min. Cells were then fixed and stained with different antibodies as indicated, and analyzed by imaging flow cytometry. (**A**) Images of T cell–tumor cell pairs. Immune synapses were highlighted by the enrichment of F-actin (blue). pLPL and CD57 distributions were marked in green and red, respectively. (**B**) Actin-enriched synapse regions were masked. Signal intensities of CD57 and pLPL were compared with those of actin and the log-transformed Pearson’s Correlation Coefficients were shown as similarity between signals. (**C**) Number of cells that had actin-accumulated synapses and pLPL/CD57 enrichment in synapses. Statistical analyses were done by ANOVA. *: *p* < 0.05; **: *p* < 0.01; ***: *p* < 0.001.

As shown in Figure 6A, the immunological synapses were clearly indicated by the enrichment of F-actin (colored in blue) between T cells and tumor cells. Phosphorylated L-plastin (pLPL), a key regulator of immunological synapse expressed in T cells [28,29], was also strongly enriched in the synapses (colored in green), indicating CAR-T cell costimulation by the tumor cells. However, the fluorescence intensity of CD57 (colored in red) near the immune synapses was lower compared with other parts of the cell membrane. We calculated the signal similarity between actin, pLPL and CD57 (Figure 6B). As expected, pLPL had a positive similarity with actin, which confirmed their colocalization in the synapses. However, CD57 had a negative similarity with actin and a similarity near zero with pLPL, strongly suggesting that CD57 was excluded from immune synapses. We further analyzed if pLPL or CD57 were enriched in the actin-marked synapses (Figure 6C). In many cells with actin-marked immune synapses, pLPL colocalized within the synapse, but almost no colocalization of CD57 was observed. Taken together, CD57 expression on HER2-CAR-T cells disappeared after antigen exposure upon coculture with tumor cells, but it did not localize in the immune synapse between CAR-T cells and tumor cells.

### 3.8. HER2-CAR-T Cells Cultivated with IL-7+15 or IL-2+7+15 Demonstrated Superior Cytotoxicity and Enhanced Production of Antitumor Cytokines upon Repetitive Tumor Challenge Compared to IL-2 Alone

**Figure 7:**
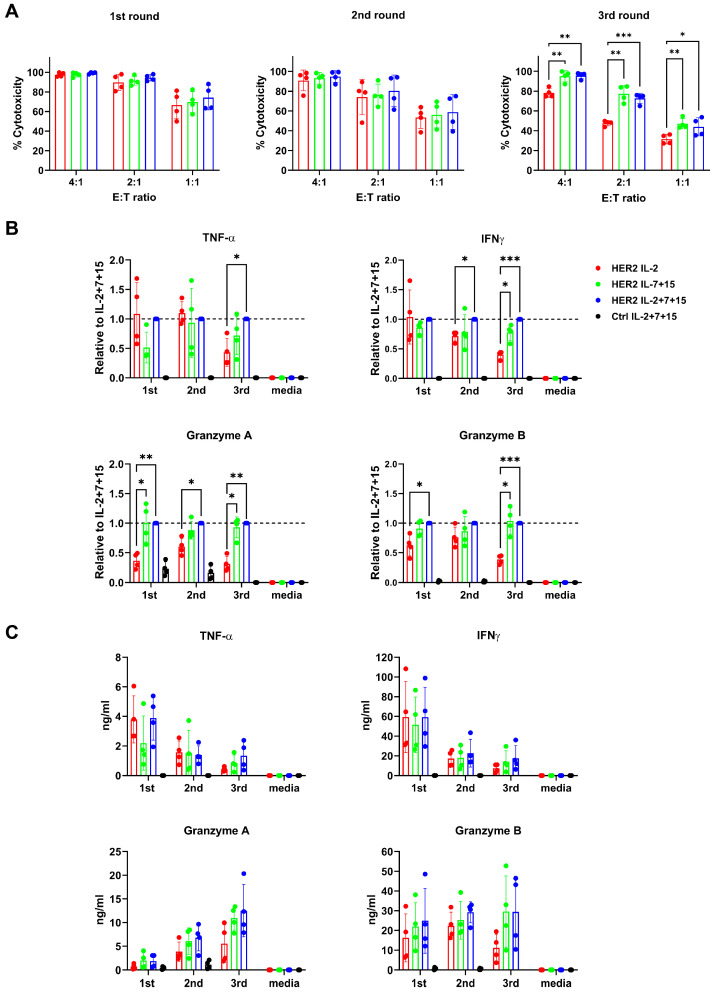
After repetitive tumor challenge, HER2-CAR-T cells cultivated with IL-2+7+15 demonstrated superior cytotoxicity and enhanced production of antitumor cytokines, compared to IL-2 alone. (**A**) HER2-CAR-T cells from each round of repetitive tumor killing assays were co-incubated with tumor cells at different effector–target (E:T) ratios for 24 h to conduct cytotoxicity assays. Remaining live tumor cells were identified by live/dead staining and HER2 expression measured by flow cytometry. (**B**,**C**) Repetitive CAR-T cell tumor cell coculture (E:T ratio 2:1). The cytokines in supernatants of each treatment were determined 6 h after each round. (**B**) Cytokine concentrations from IL-2- or IL-7+15-treated cocultures from individual donors were normalized. The IL-2+7+15 group was set as 1. Media: tumor cell culture media control. Statistical analysis was done by one-way ANOVA. *: *p* < 0.05; **: *p* < 0.01; ***: *p* < 0.001. (**C**) Absolute concentration of cytokines in 6 h culture supernatants.

In previous repetitive tumor exposure assays, we have demonstrated the superior expansion of IL-2+7+15-empowered HER2-CAR-T cells (compare Figure 3A). Such persistence in the expansion of CAR-T cells would be necessary to enhance their antitumor function in vivo. To test if IL-2+7+15 treatment could also enhance the cytotoxicity of CAR-T cells during repetitive tumor exposure, T cells were collected before each tumor (re-)exposure to perform individual cytotoxicity assays at different E:T ratios (4:1, 2:1, 1:1) within 24 h. No significant differences were observed after the first and second round of the repetitive tumor exposure (Figure 7A, left and middle). However, in the third round, IL-7+15- and IL-2+7+15-treated HER2-CAR-T cells exhibited significantly stronger cytotoxicity against SKBR-3 tumor cells than IL-2-treated CAR-T cells at all E:T ratios (Figure 7A, right). The most significant difference in cytotoxicity between IL-2-treated CAR-T cells and IL-2+7+15-treated CAR-T cells was observed at an E:T ratio of 2:1, where IL-2+7+15-treated CAR-T cells achieved approximately 30% higher levels of cytotoxicity compared to IL-2-treated CAR-T cells (75% vs. 45%, respectively).

To further explore the molecular mechanism of how CAR-T cells empowered by IL-7 and IL-15 killed better, the cell supernatants 6 h after tumor exposure (E:T ratio 2:1) were collected and analyzed for antitumor cytokine production of HER2-CAR-T cells. In addition, individual normalized values were calculated to minimize donor-dependent differences (Figure 7B). The respective absolute concentrations are shown in Figure 7C. Although the production of TNF-α and IFN-γ decreased by each tumor challenge (Figure 7C, upper panel), CAR-T cells cultured with IL-7+15 and IL-2+7+15 maintained higher production of these cytokines relative to IL-2-cultured CAR-T cells (Figure 7B). Unlike TNF-α and IFN-γ, the levels of granzyme A and B increased over time (Figure 7C, lower panel), except for granzyme B in IL-2-only treated CAR-T cells. Notably, CAR-T cells also expanded with IL-2+7+15 produced substantially higher amounts of all four analytes compared to IL-2-treated CAR-T cells. Specifically, there was approximately a 3-fold increase in TNF-α and IFN-γ, and approximately a 4-fold increase in granzyme A and granzyme B. IL-7+15-treated cells showed a cytokine production profile closely resembling that of the IL-2+7+15 group. The enhanced granzyme A and B secretion, in conjunction with the cytotoxicity assays shown in Figure 7A, confirmed enhanced CAR-T cell activation/cytotoxicity upon tumor cell encounter, if CAR-T cells were cultured with IL-2+7+15.

In conclusion, IL-7+15- and IL-2+7+15-treated HER2-CAR-T cells demonstrated greater cytotoxicity with improved production of antitumor cytokines.

## 4. Discussion

In this study, we systematically evaluated how combinations of common γ-chain cytokines influence the phenotype, persistence, and antitumor function of HER2-CAR-T cells during ex vivo manufacturing and repetitive tumor exposure. We demonstrate that supplementation with IL-7 and IL-15, either in combination (IL-7+15) or together with low-dose IL-2 (IL-2+7+15), substantially improves CAR-T cell expansion, durability, and effector function compared with conventional IL-2-based protocols. Notably, IL-7+15 and IL-2+7+15 treatment preserved stem cell-like features prior to antigen encounter and sustained cytotoxicity with enhanced cytokine production during multiple rounds of tumor challenge.

Across most phenotypic and functional parameters, IL-7+15- and IL-2+7+15-treated cells performed comparably, suggesting that IL-7 and IL-15 are the dominant drivers of the favorable CAR-T cell phenotype observed here, while low-dose IL-2 may primarily support expansion without overriding the differentiation program induced by IL-7 and IL-15. The application of γ-chain cytokines beyond IL-2 has become increasingly prominent in CAR-T manufacturing. Two major strategies have emerged: exogenous cytokine supplementation during ex vivo expansion [19,22,23,30] and genetic engineering approaches co-expressing interleukins or their receptors [20,21,31]. While genetic approaches provide sustained signaling, they carry risks of cytokine release syndrome due to constitutive or tumor-induced cytokine production [31]. Our manufacturing-based approach circumvents these concerns by limiting interleukin exposure to the ex vivo phase. Notably, we used conservative interleukin concentrations, particularly reducing IL-2 to 20 U/mL versus the typical 50–500 U/mL in conventional protocols. Despite this, IL-2+7+15-treated cells demonstrated robust proliferation, cytotoxicity, sustainability and enriched stem cell memory populations. These findings demonstrate that the judicious combination of low-dose IL-2 with IL-7 and IL-15 preserves the physiological role of IL-2 in supporting T cell activation while harnessing the memory-promoting and anti-exhaustion benefits of IL-7 and IL-15, offering a clinically translatable manufacturing strategy that improves both the phenotypic quality and functional sustainability of CAR-T cell products. Note that a comprehensive functional comparison of IL-2+7 and IL-2+15 conditions was beyond the predefined scope of this study, which was specifically designed to evaluate the additive effects of the triple IL-2+7+15 combination. Nevertheless, future studies should address the relative contribution of these dual combinations in greater depth.

We observed a significant, albeit modest, upregulation of PD1 specifically in CD8^+^ CAR-T cells following IL-2+7+15 cultivation during CAR-T cell expansion. In contrast, the upregulation of TIM3 and LAG3 induced by IL-2+7+15 was more pronounced and occurred similarly in both CD4^+^ and CD8^+^ subsets. These findings suggest that cytokine-mediated modulation of differentiation and activation states may differentially affect the CD8^+^ and CD4^+^ compartments. Importantly, caution should be exercised when interpreting the upregulation of inhibitory receptors such as PD1, TIM3, and LAG3 as direct evidence of T cell exhaustion. The expression of these molecules is dynamically regulated by the strength and duration of T cell stimulation, and is also controlled by transcriptional programs involving key signaling molecules including STAT5, T-bet, and EOMES [32]. True T cell exhaustion typically arises under conditions of chronic antigen stimulation, and is defined not solely by inhibitory receptor expression, but by a combination of characteristic transcriptional signatures, impaired proliferative capacity, and compromised effector function [33]. CAR-T cells cultured with IL-7+15 or IL-2+7+15 retained robust proliferative capacity and sustained cytotoxic activity during repetitive tumor challenge assays, indicating that the observed inhibitory receptor expression does not correspond to a state of functional exhaustion. Therefore, the increased expression of TIM3 and LAG3 observed in our study more likely reflects a state of activation rather than terminal exhaustion.

A key observation of our study is the pronounced proliferative advantage conferred by IL-7 and IL-15 after the early expansion phase. While CAR-T cells expanded similarly across cytokine conditions during the first week after activation, IL-7+15- and IL-2+7+15-treated cells exhibited markedly enhanced proliferation between days 8 and 15. This finding is consistent with the established roles of IL-7 and IL-15 in promoting T cell survival, homeostatic proliferation, and resistance to activation-induced cell death. Importantly, this proliferative advantage has translated into superior persistence during repetitive antigen exposure, a condition that more closely mimics the chronic stimulation encountered by CAR-T cells in solid tumors.

Our transcriptional analysis further revealed that CAR-T cells expanded with IL-2+7+15 displayed increased expression of genes associated with stem cell memory and long-term persistence, including *TCF7* (TCF-1), *FOXO1*, and *CD27*. The enrichment of this transcriptional signature supports the notion that IL-7 and IL-15 help preserve a less differentiated, self-renewing phenotype during manufacturing. Although tumor antigen exposure rapidly drove differentiation toward effector memory states across all conditions, CAR-T cells generated with IL-7 and IL-15 retained superior expansion and functional capacity, suggesting that early preservation of stem-like qualities confers durable benefits even after differentiation.

We observed enriched CD57^+^ populations in CAR-T cells cultured with IL-15-containing regimens, particularly in CD8^+^ T cells. CD57 has been debated as a functional marker. Historically established as marking immunosenescence and terminal differentiation based on reduced proliferative capacity and shortened telomeres in chronic viral infections [17,26,34], this view has been challenged by demonstrations of retained proliferative potential under specific conditions [35,36], and further studies have revealed that CD57^+^ memory T cells retain proliferative capacity [37]. Our findings contribute in two ways. Firstly, consistent with previous reports [38], IL-15 supplementation during ex vivo culture induces CD57 expression independently of chronic antigen exposure in vivo. Secondly, CD57 expression was dramatically downregulated following tumor antigen stimulation, persisting even without continued tumor exposure. This occurred in both CD57^hi^ and bulk CAR-T cell populations, suggesting active regulation rather than selective depletion. The reversibility of CD57 expression challenges its interpretation as a fixed terminal differentiation marker and instead suggests a dynamic, activation-responsive phenotype in ex vivo-manufactured CAR-T cells. We propose that CD57^+^ cells generated through IL-15 supplementation during manufacturing may differ functionally from those in chronic inflammation in vivo, where sustained IL-15 may drive stable senescence [34]. Importantly, CD57^+^ cells did not accumulate during repetitive tumor killing despite the continuous presence of IL-15, suggesting that antigen stimulation limits or reverses CD57 expression in this context. Imaging flow cytometry further showed that CD57 is not enriched at the immunological synapse. Whether synapse formation contributes to CD57 internalization, shedding, or proteolytic cleavage remains an open question and would require dedicated experiments beyond the scope of the present study. Nevertheless, the observation that CD57 expression decreases following antigen exposure indicates that this phenotype is dynamically regulated. Together, our findings suggest that CD57^+^ CAR-T cells retain the capacity for antigen-induced phenotypic remodeling, supporting the concept of functional plasticity rather than irreversible senescence.

Treatment with IL-2+7+15 did not significantly increase the overall proportions of CD4^+^ or CD8^+^ T cells during the initial CAR-T cell expansion phase. However, IL-2+7+15 treatment increased the frequency of CD4^+^ T_scm_ cells, but not CD8^+^ T_scm_, compared with IL-2 treatment alone. Notably, during repetitive tumor challenges, IL-2+7+15 treatment preferentially promoted CD4^+^ T cells. This enrichment of CD4^+^ cells may be clinically relevant, as a 1:1 CD4^+^/CD8^+^ ratio has been associated with superior long-term antitumor responses in CAR-T therapies [39,40]. Moreover, beyond direct cytotoxicity, CD4^+^ CAR-T cells fulfill pivotal supportive functions. They maintain the proliferation and killing capacity of stem-like CD8^+^ CAR-T cells through their helper functions [30] and serve as major producers of IFN-γ, particularly within Th1 subsets. The mechanisms by which IL-2+7+15 preferentially promotes CD4^+^ T_scm_ enrichment and expansion remain unclear and represent an important area for future investigation. Although less studied than their CD8^+^ counterparts, CD4^+^ T cells can also upregulate inhibitory receptors upon persistent antigen stimulation. This can result in the subsequent compromise of effector function and proliferation [41]. Such an impairment can be reversed by anti-PD1 treatment [42], thereby underscoring the functional significance of PD1 expression on CD4^+^ T cells. A notable finding of our study was the observation that, subsequent to repeated exposure to tumor cells, IL-2+7+15-treated CAR-T cells expressed significantly less PD1 than IL-2-treated CAR-T cells. This finding implies that the effector function of these cells may be less downregulated by PDL1 expressed on tumor cells, which could provide a potential explanation for the sustained effector function of IL-2+7+15-treated CAR-T cells as compared to IL-2-treated CAR-T cells. Together, the IL-2+7+15 protocol has been shown to have several advantageous properties, including the enrichment of CD4^+^ T cells, the suppression of PD1 expression, and the enhancement of IFN-γ production. These properties suggest a synergistic mechanism that may enhance the efficacy of CAR-T cell therapy through both optimized cellular composition and enhanced immunomodulatory function.

In conclusion, our study demonstrates that incorporating IL-7 and IL-15 into CAR-T cell manufacturing addresses multiple determinants of therapeutic success: enhanced proliferation, enriched stem cell memory populations, optimized CD4^+^/CD8^+^ ratios, and augmented multi-modal cytotoxic mechanisms. These improvements were achieved using conservative cytokine concentrations, ensuring clinical translatability while circumventing safety concerns of genetic engineering approaches. While this study focuses on HER2-targeted CAR-T cells, the fundamental benefits of IL-7 and IL-15 supplementation are likely broadly applicable across CAR-T platforms. Our findings provide both mechanistic insights and a practical framework for next-generation manufacturing protocols that could enhance CAR-T cell therapeutic efficacy across cancer types, offering a readily implementable strategy to advance CAR-T therapy toward broader clinical applications.

## 5. Conclusions

This study demonstrates that incorporating IL-7 and IL-15 into HER2-CAR-T cell manufacturing significantly enhances expansion, persistence, and antitumor activity compared with conventional IL-2-based protocols. The IL-2+7+15 combination enhanced proliferation—particularly of CD4^+^ cells during repetitive tumor challenge—enriched stem cell memory (T_scm_) populations, and sustained cytotoxicity through multiple rounds of tumor challenges. Importantly, these benefits were achieved using conservative IL-2 concentrations through ex vivo cytokine supplementation, thereby avoiding the safety concerns associated with genetic interleukin co-expression strategies.

We further found that IL-15-induced CD57^+^ CAR-T cells retained full proliferative and cytotoxic capacity comparable to their CD57^−^ counterpart, despite the historical characterization of CD57 as a marker of immunosenescence. The dynamic downregulation of CD57 upon antigen stimulation suggests phenotypic plasticity in HER2-CAR-T cells and challenges rigid interpretations of differentiation states derived from chronically stimulated in vivo settings.

Although our in vitro model demonstrates clear benefits of IL-7 and IL-15 during CAR-T cell generation and repetitive tumor killing, in vivo validation in solid tumor models will be required to confirm therapeutic efficacy and long-term persistence.

Collectively, this work provides both mechanistic insight and a practical, readily implementable manufacturing strategy that may help advance CAR-T cell therapy toward more effective treatment of solid tumors. Because this approach relies on ex vivo cytokine supplementation rather than genetic modification, it could be readily incorporated into existing CAR-T cell manufacturing workflows.

## Data Availability

The original contributions presented in this study are included in the article/Supplementary Material. Further inquiries can be directed to the corresponding authors.

## Supplementary Materials

The following supporting information can be downloaded at: https://www.mdpi.com/article/10.3390/cells15060547/s1, Figure S1: exhaustion phenotype of CD4^+^ CAR-T cells; Figure S2: gating strategy of memory status of CAR-T cells; Figure S3: T_em_ and T_emra_ population of CD4^+^ and CD8^+^ cells during the CAR-T cell expansion; Figure S4: memory phenotype of CD4^+^ and CD8^+^ CAR-T cells without exposure to tumor cells; Figure S5: CD57 expression level of CD4^+^ and CD8^+^ HER2-CAR-T cells with different interleukin treatments during expansion; Figure S6: the decrease in CD57 levels on HER2-CAR-T cells after tumor exposure did not recover after culture in the absence of antigen for 4 days; Figure S7: CD57^+^ CAR-T cells survived after antigen stimulation and kept proliferating; Figure S8: GLUT1 expression did not differ in CD57^hi/lo^ population; Table S1: nCounter Exhaustion panel gene expression profile IL-2+7+15 CAR-T cells versus IL-2 CAR-T cells.
