# Supplementary material for: Empowerment of CAR-T Cells by IL-7 and IL-15 Boosts Their Efficacy Against HER2-Positive Tumors with Enhanced Expansion and Persistence
Source: Cells. 2026 Mar 19;15(6):547. doi: 10.3390/cells15060547 (PMC13026050; doi:10.3390/cells15060547)
Supplement: Supplementary file 1 [file cells-15-00547-s001.zip › Supplementary Figures.pdf]

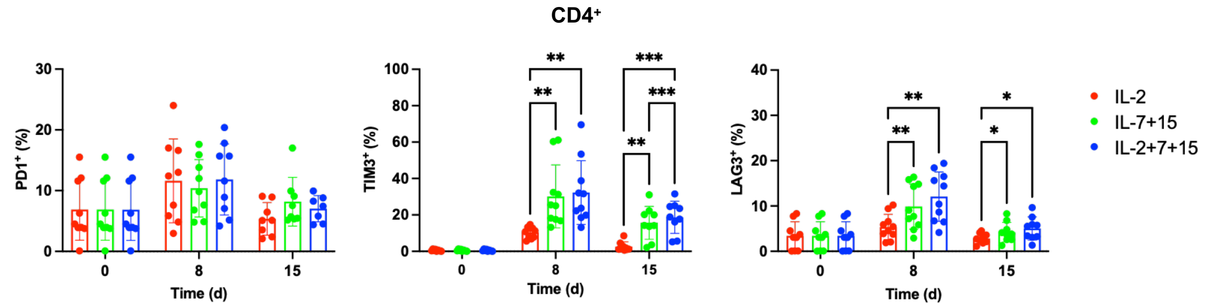

Figure S1. Exhaustion phenotype of $CD4^+$ CAR-T cells. On day 8 and day 15 after initial activation, HER2-CAR-T cells were stained and measured for the expressions of PD1, TIM3 and LAG3.

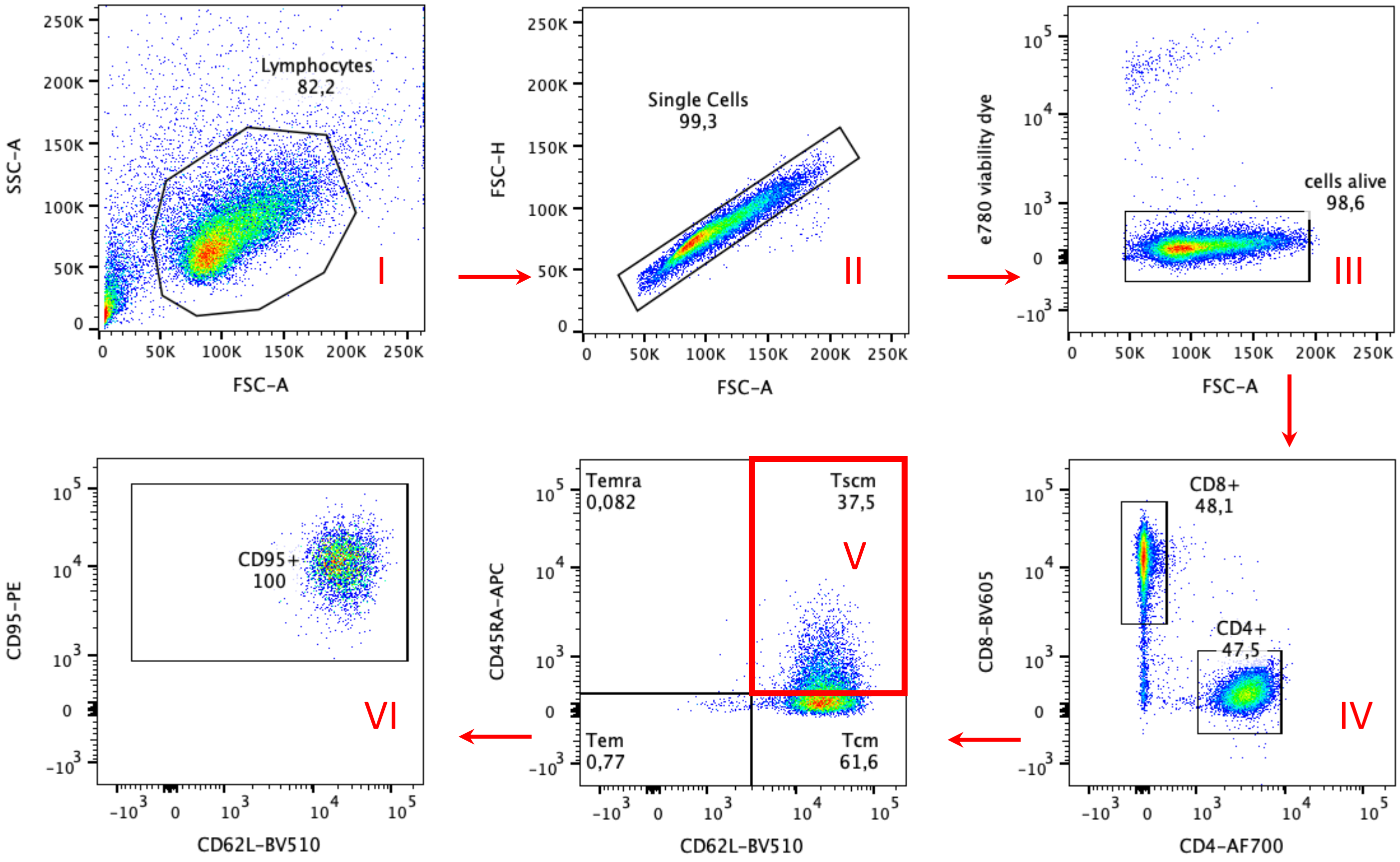

Figure S2. Gating strategy of memory status of CAR-T cells. 8 days after initial activation, CAR-T cells were stained for CD4, CD8, CD62L, CD45RA, CD95 and viability. Cells were first selected from debris based on FSC-A and SSC-A features (region I). Then, single cells were selected based on FSC-A and FSC-H features (region II). The dead cells were excluded from the living cells (region III) by e780 viability staining. Next, CAR-T cells were discriminated by CD4 and CD8 staining (region IV). Their memory status were determined by CD62L and CD45RA staining (region V), here $CD4^+$ cells were taken as example. To distinguish T_{scm} from $CD95^-$ naïve T cells, CD95 was further stained (region VI).

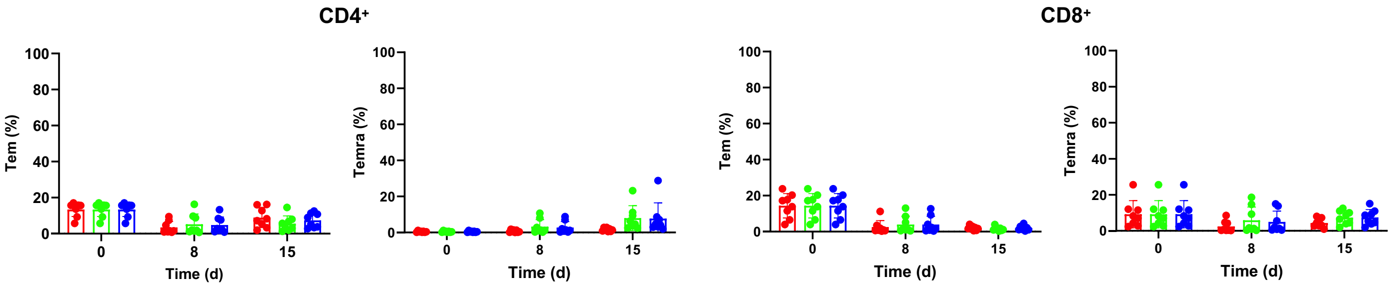

Figure S3. T_{em} and T_{emra} population of $CD4^+$ and $CD8^+$ cells during the CAR-T cell expansion. On day 8 and day 15 after initial activation, HER2-CAR-T cells were stained and measured for the memory phenotype. T_{em}: $CD62L^-CD45RA^-$; T_{emra}: $CD62L^-CD45RA^+$.

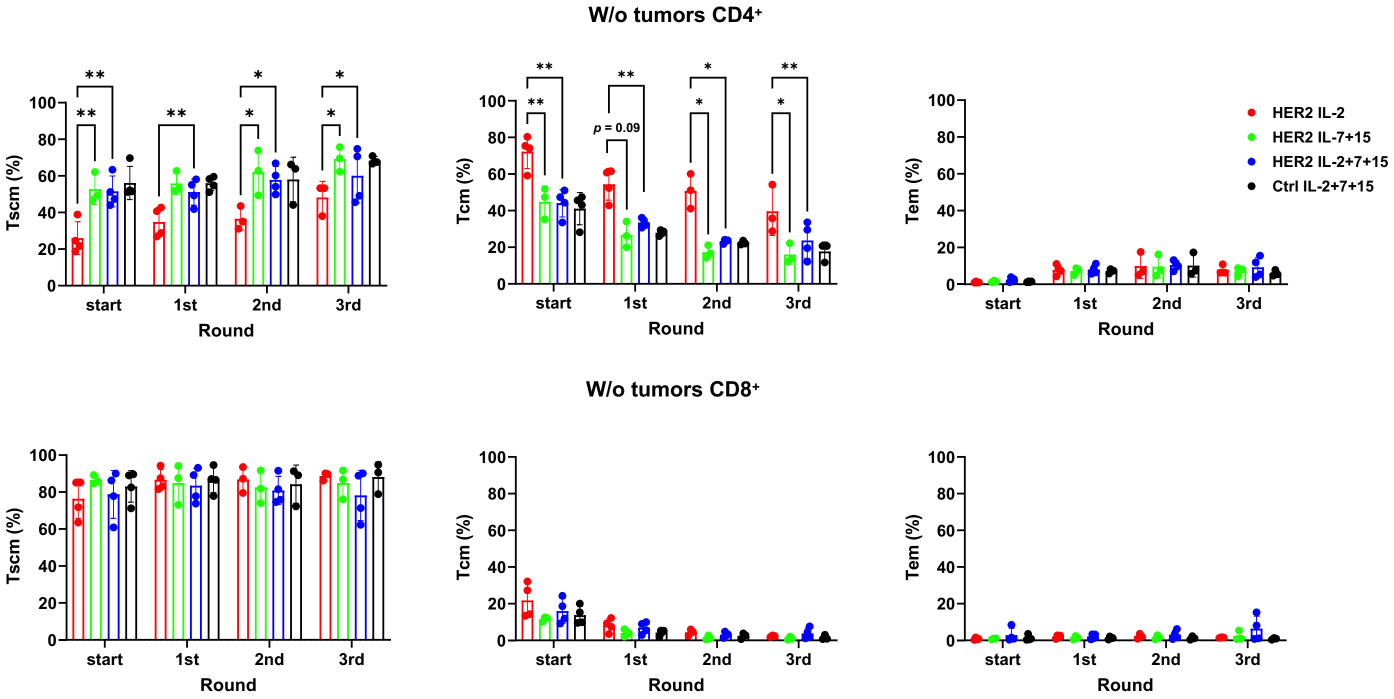

Figure S4. Memory phenotype of CD4$^+$ and CD8$^+$ CAR-T cells without exposure to tumor cells. As a control**,** 9 days after initial activation (start), when repetitive tumor killing assays were started, CAR-T cells were also cultured without tumor cells. They were measured for memory phenotype at the same time when the CAR-T cells of repetitive tumor killing assays were measured.

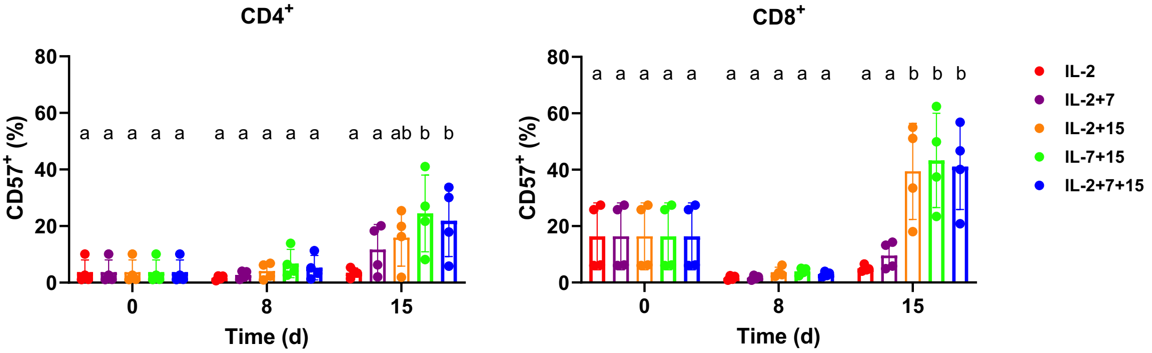

Figure S5. CD57 expression level of CD4$^+$ and CD8$^+$ HER2-CAR-T cells with different interleukin treatment during expansion. HER2-CAR-T cells cultured with different interleukin combinations were measured for the expression of CD57 during CAR-T cell expansion. The statistical analysis was done by matched ANOVA by donors. Means with different letters are significantly different ($p < 0.05$).

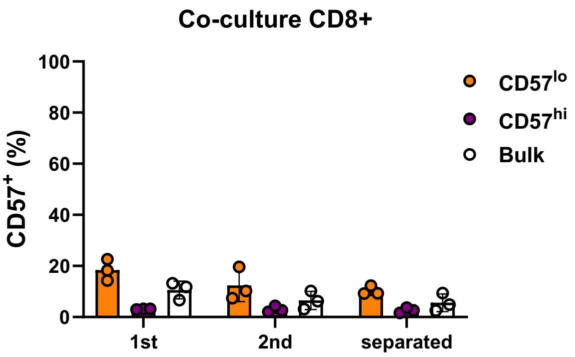

Figure S6. The decrease in CD57 levels on HER2-CAR-T cells after tumor exposure did not recover after culture in the absence of antigen for 4 days. IL-2+7+15 HER2-CAR-T cells were positively selected on day 15 after activation. On day 16, The positively selected CD57^{hi} CAR-T cells, the remaining CD57^{lo} cells and bulk CAR-T cells were proceeded to a tumor killing assay. After the first exposure, the CAR-T cells were cultured without tumor cells for 4 days and measured for CD57 expression.

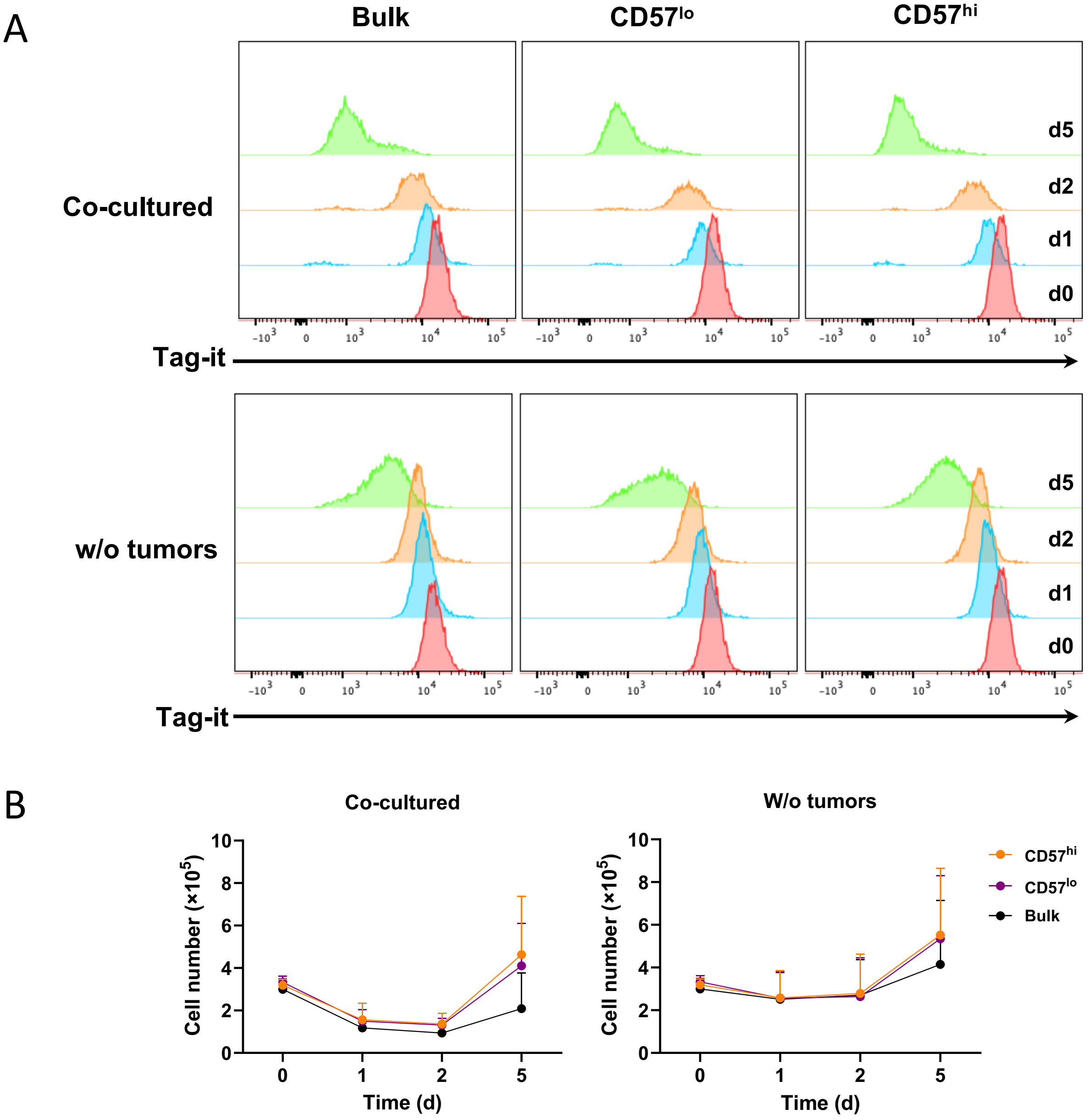

Figure S7. CD57⁺ CAR-T cells survived after antigen stimulation and kept proliferating. (**A**) CD57^hi^, CD57^lo^ and bulk CAR-T cells, as described before, were stained with Tag-it® proliferation dye before co-culture with tumor cells to track their cell divisions. (**B**) Cell counts for CAR-T cells between day 0 and day 5 of coculture with tumor cells.

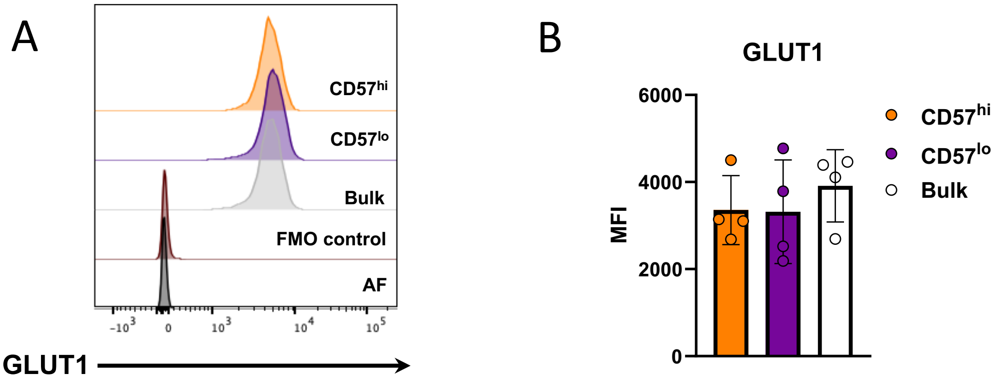

Figure S8. GLUT1 expression did not differ in CD57$^{hi/lo}$ population. After the CD57 positive selection, CAR-T cells were analyzed for GLUT1 expression by flow cytometry. (**A**) Representative histogram of GLUT1 expression in CD57$^{hi/lo}$ and bulk HER2-CAR-T cells with IL-2+7+15. (**B**) Mean fluorescence intensity (MFI) of GLUT1 expression on CAR-T cells.
